# Inferring miRNA-disease associations using collaborative filtering and resource allocation on a tripartite graph

**DOI:** 10.1186/s12920-021-01078-8

**Published:** 2021-11-17

**Authors:** Van Tinh Nguyen, Thi Tu Kien Le, Tran Quoc Vinh Nguyen, Dang Hung Tran

**Affiliations:** 1 Faculty of Information Technology, Hanoi University of Industry, Hanoi, Vietnam; 2 Faculty of Information Technology, Hanoi National University of Education, Hanoi, Vietnam; 3 Faculty of Information Technology, The University of Da Nang - University of Science and Education, Da Nang, Vietnam

**Keywords:** Infer miRNA-disease associations, miRNA-disease-lncRNA tripartite graph, Collaborative filtering algorithm, Resource allocation algorithm, Recommender systems

## Abstract

**Background:**

Developing efficient and successful computational methods to infer potential miRNA-disease associations is urgently needed and is attracting many computer scientists in recent years. The reason is that miRNAs are involved in many important biological processes and it is tremendously expensive and time-consuming to do biological experiments to verify miRNA-disease associations.

**Methods:**

In this paper, we proposed a new method to infer miRNA-disease associations using collaborative filtering and resource allocation algorithms on a miRNA-disease-lncRNA tripartite graph. It combined the collaborative filtering algorithm in CFNBC model to solve the problem of imbalanced data and the method for association prediction established multiple types of known associations among multiple objects presented in TPGLDA model.

**Results:**

The experimental results showed that our proposed method achieved a reliable performance with Area Under Roc Curve (AUC) and Area Under Precision-Recall Curve (AUPR) values of 0.9788 and 0.9373, respectively, under fivefold-cross-validation experiments. It outperformed than some other previous methods such as DCSMDA and TPGLDA. Furthermore, it demonstrated the ability to derive new associations between miRNAs and diseases among 8, 19 and 14 new associations out of top 40 predicted associations in case studies of Prostatic Neoplasms, Heart Failure, and Glioma diseases, respectively. All of these new predicted associations have been confirmed by recent literatures. Besides, it could discover new associations for new diseases (or miRNAs) without any known associations as demonstrated in the case study of Open-angle glaucoma disease.

**Conclusion:**

With the reliable performance to infer new associations between miRNAs and diseases as well as to discover new associations for new diseases (or miRNAs) without any known associations, our proposed method can be considered as a powerful tool to infer miRNA-disease associations.

**Supplementary Information:**

The online version contains supplementary material available at 10.1186/s12920-021-01078-8.

## Background

MicroRNA (miRNA) is a small RNA, about 22–26 nucleotides, which belongs to the noncoding RNA class [1]. Recent researches have shown that miRNAs are involved in many crucial biological processes like cell differentiation, proliferation, signal transduction, viral infection, and so on [2]. Identifying miRNA-disease associations could not only help us understand disease mechanism at miRNA level but also facilitate us in detecting disease biomarkers and discovering drugs for disease diagnosis, treatment, prognosis, and prevention. It has been confirmed that the dysregulations of the miRNAs are associated with the development and progression of various complex human diseases [3–6]. Until now, there are only a few known miRNA-disease associations in comparison with the number of newly discovered miRNAs. It is also tremendously expensive and time-consuming to do biological experiments to verify miRNA-disease associations. Therefore, expanding effective and outstanding computational methods to predict potential miRNA-disease associations is urgently needed and is attracting many computer scientists in recent years [7].

Recently, various computational methods to forecast possible miRNA-disease associations have been developed. For example, Liu et al. [8] proposed PBMDA prediction model which integrated known human miRNA-disease associations, miRNA functional similarity, disease semantic similarity and Gaussian interaction profile kernel similarity for miRNAs and diseases. They constructed a heterogeneous graph and further adopted depth-first search algorithm to figure out probable miRNA-disease associations. Chen et al*.* [9] presented a model called Graphlet Interaction for miRNA-Disease Association prediction (GIMDA) to predict miRNA-disease associations by measuring the graphlet interaction among miRNAs and among diseases. Graphlet is a type of subgraph with a few connections in a large network. GIMDA achieved a decisive performance but it was significantly time-consuming. Liang et al*.* [10] proposed a miRNA-disease association prediction method based on adaptive multi-view multi-label learning (AMVML). It learned a new affinity graph for miRNAs and diseases from multiple data sources. However, the integration of unreliable similarity matrices might weaken its overall prediction accuracy. The above mentioned methods for predicting miRNA-disease associations strongly relied on known human miRNA-disease associations. Most of existing methods need to use the similarity matrices such as the disease semantic similarity matrix and miRNA functional similarity matrix but they are not directly related to the miRNA-disease associations [11]. Besides, they have to deal with the problem of sparse similarity matrices which affected the prediction accuracies [12]. One other problem is that the miRNA-target interactions usually have a high rate of false-positive and false-negative [9, 13].

In fact, diseases are caused by the disturbance of a complex of interacting multiple biomolecules rather than the abnormity of a single biomolecule. The functionally dependent molecular components in human cells form a complex biological network, in which lncRNAs and proteins are important parts of human tissues and cells. It is the reason that some computational methods have recently based on multiple types of known associations or interactions among multiple objects to predict potential miRNA-disease associations. For example, Zhao et al*.* [7] developed a computational method based on a distance correlation set to predict miRNA-disease associations (DCSMDA) by integrating known lncRNA-disease associations, known miRNA-lncRNA associations, disease semantic similarity, and various lncRNA and disease similarity measures. DCSMDA did not require known miRNA-disease associations but it required the calculation of various similarity matrices and its performance depended on the pre-given threshold parameter. Mørk et al*.* [14] relied on known miRNA–protein associations and known protein–disease associations to infer miRNA–disease associations. Marissa Sumathipala and Weiss [15] integrated miRNA-gene, protein–protein, and gene-disease network information into a multi-level complex network to predict and prioritize biologically relevant miRNAs for diseases. Ji et al*.* [16] constructed a heterogeneous information network by integrating the known associations among lncRNAs, drugs, proteins, diseases, and miRNAs. They further employed the network embedding method which learned graph representations with global structural information to predict miRNA-disease associations. In general, the computational methods for predicting miRNA-disease associations based on multiple types of known associations among multiple objects are usually helpful for improving prediction accuracy. However, the number of known associations among biological objects is very limited in comparison with the number of objects in each type. Therefore, once again, these models have to be considered with the sparsity data problem.

In recent years, a variety of recommender systems have been developed to increase the association prediction reliability based on collaborative filtering methods. These methods rely on prior actions to predict user-item relationships to solve the problem of scarce known associations among different objects [17, 18]. Up to date, recommender algorithms have been appended into some computational models of prediction to identify different potential disease related biological objects. For example, Yu et al*.* [19] proposed a collaborative filtering model for lncRNA-disease association prediction based on the Naïve Bayesian classifier. Shen et al*.* [2] predicted miRNA-disease association with Collaborative Matrix Factorization model which caused bias to miRNAs with more known associated diseases. Li et al*.* [11] presented a collaborative filtering-based miRNA-disease association prediction model (CFMDA) to predict miRNA-disease association. CFMDA was straight and robust by considering a minimal amount of related information and no tunable parameters were defined. However, CFMDA’s association prediction performance was subjective because it only relies on miRNA-disease associations to execute predictions.

To solve the sparsity data problem and to take advantages of the integration of multiple types of known associations among multiple objects in improving prediction accuracy, in this paper, we proposed a new method to infer miRNA-disease associations using collaborative filtering and resource allocation algorithms on a tripartite graph. Our method is inspired by combining the collaborative filtering algorithm in CFNBC model introduced by Yu et al. [19] to solve the problem of imbalanced data and the method for association prediction established multiple types of known associations among multiple objects presented in TPGLDA model which introduced by Ding et al*.*[20] and the model in our former study [21]. Firstly, we constructed a tripartite graph which based on the known miRNA-disease associations, the known lncRNA-disease associations, and the known miRNA-lncRNA interactions. Secondly, we used a collaborative filtering algorithm to recommend miRNAs for lncRNAs and diseases, respectively. Next, we employed a resource allocation algorithm to infer miRNA-disease associations. Finally, we ranked all candidate miRNAs for each disease in descending order to suggest associations between miRNAs and diseases for further giving the evidence in the future. Our method achieved a trustworthy prediction performance under fivefold-cross-validation experiments with an Area Under Roc Curve (AUC) averaged value of 0.9788 and an Area Under Precision-Recall Curve (AUPR) averaged value of 0.9373. It is outperformance in comparison to several previous methods such as the DCSMDA [7] and the TPGLDA [20].

## Methods

### Materials

In this paper, we used datasets which came from the study of Zhao et al. [7]. We downloaded and used the Additional files 1, 2, 3, 4, and 5 from this study. These datasets contain 190 diseases, 111 lncRNAs and 264 miRNAs as described as follows:

#### Known lncRNA-miRNA associations

The known lncRNA-miRNA associations were collected from the starBasev2.0 [22] in February, 2017 and provided the most comprehensive experimentally confirmed lncRNA-miRNA interactions based on large-scale CLIP-Seq data. After eliminating duplicate values and erroneous data and also removing lncRNAs not included in DS2 dataset, we obtained the DS1 dataset which contains 1880 known lncRNA-miRNA associations.

#### Known lncRNA-disease associations

The known lncRNA-disease associations were collected from 8842 known disease-lncRNA associations in the MNDR database [23] and 2934 known disease-lncRNA associations in the LncRNADisease database [24]. After eliminating diseases without any MeSH descriptors because the disease names came from two different databases, merging the diseases with the same MeSH descriptors and removing the lncRNAs which were not included in the lncRNA-miRNA dataset (DS1), 936 known associations between diseases and lncRNAs (DS2) remained.

#### Known disease-miRNA associations

The known human miRNA-disease associations were downloaded from the HMDD V2.0 database [25]. This dataset (DS3) contains 3252 quality miRNA-disease associations after we eliminated the duplicate associations and miRNA-disease associations involving with other diseases or lncRNAs which were not contained in the DS1 or DS2 datasets.

### Method overview

**Fig. 1:**
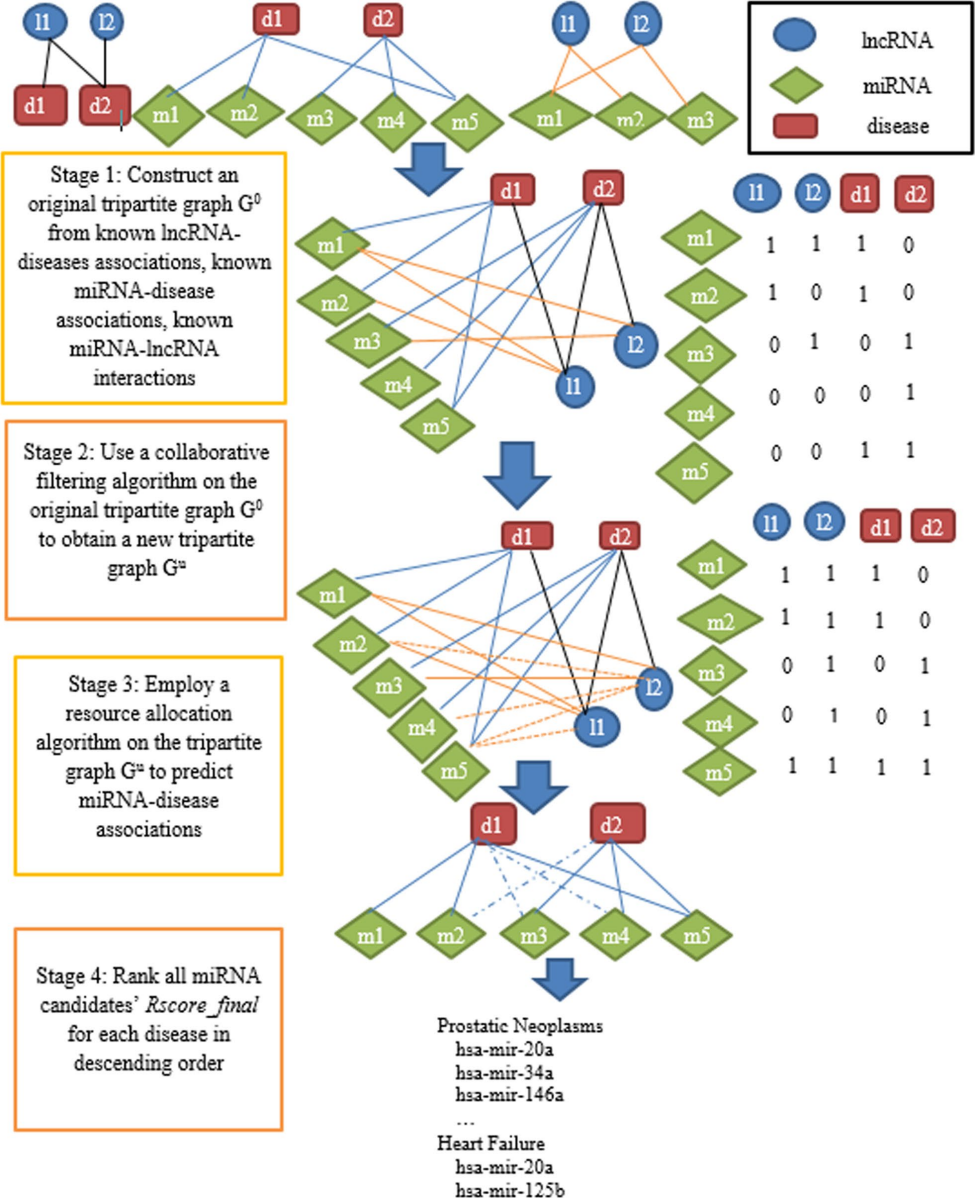
The flowchart of the proposed method

In this paper, we proposed a new method to infer miRNA-disease associations. The flowchart of the proposed method is illustrated in Fig. 1. Generally, our proposed method contains four main stages. At the first stage, we constructed a tripartite graph G^0^ based on known miRNA-disease associations, known lncRNA-disease associations, and known miRNA-lncRNA interactions. The tripartite graph G^0^ is represented by three adjacency matrices: *A*^*0*^_*MD,*_* A*^*0*^_*ML*_ and *A*^*0*^_*DL*_ where *A*^*0*^_*MD*_ is the adjacency matrix between miRNAs and diseases, *A*^*0*^_*ML*_ is the adjacency matrix between miRNAs and lncRNAs, *A*^*0*^_*DL*_ is the adjacency matrix between diseases and lncRNAs. During the second stage, to solve the imbalance data problem, we employed a collaborative filtering algorithm on the tripartite graph G^0^ to obtain a tripartite graph G^u^. The tripartite graph G^u^ is represented by three adjacency matrices: *A*^*u*^_*MD,*_* A*^*u*^_*ML*_ and *A*^*0*^_*DL*_ where *A*^*u*^_*MD,*_* A*^*u*^_*ML*_ are the adjacency matrices obtained by updating *A*^*0*^_*MD*_ and *A*^*0*^_*ML*_ after using collaborative filtering algorithm. The tripartite graph G^u^ is used in a resource allocation algorithm at the third stage to calculate final resource score *(Rscore_final)* of miRNA candidates for each disease. At the final stage, we ranked all miRNA candidates’ *Rscore_final* for each disease in descending order so that the candidate with greater *Rscore_final* will have higher possibility to be verified in the future.

### Construction of a tripartite graph G^*0*^

Inspired by previous studies [19, 20] to infer lncRNA-disease associations by using a tripartite graph, in this paper, we firstly construct a miRNA-disease-lncRNA tripartite graph G^0^ as follows:

#### Construction of known miRNA-disease association graph

Let *M* = *{m*_*k*_*; k* = *1,…,n*_*m*_*}* denotes the set of miRNAs, *D* = *{d*_*j*_*; j* = 1,…, *n*_*d*_*}* denotes the set of diseases where *n*_*m*_*, n*_*d*_ represent the number of miRNAs and diseases, respectively. We build a MD^0^ graph based on the known miRNA-disease associations. The MD^0^ graph is represented by a matrix *A*^*0*^_*MD*_ which is the adjacency matrix of known miRNA-disease associations. The entity *A*^*0*^_*MD*_*(m*_*k*_*, d*_*j*_*)* is the element in *k*th row and *j*th column of *A*^*0*^_*MD*_, and *A*^*0*^_*MD*_*(m*_*k*_*, d*_*j*_*)* = *1* if miRNA m_k_ is associated with disease *d*_*j*_, otherwise, *A*^*0*^_*MD*_*(m*_*k*_*, d*_*j*_*)* = *0*.

#### Construction of known miRNA-lncRNA interaction graph

In the same way, let *M* = *{m*_*k*_*; k* = *1,…,n*_*m*_*}* denotes the set of miRNAs, *L* = *{l*_*i*_*; i* = *1,…, n*_*l*_*}* denotes the set of lncRNAs where *n*_*m*_*, n*_*l*_ represent number of miRNAs and lncRNAs, respectively. We can obtain *ML*^*0*^ graph and *A*^*0*^_*ML*_ matrix. *ML*^*0*^ graph is built on known miRNA-lncRNA interactions. *A*^*0*^_*ML*_ is the adjacency matrix of known miRNA-lncRNA interactions. The entity *A*^*0*^_*ML*_*(m*_*k*_*, l*_*i*_*)* is the element in *k*th row and *i*th column of *A*^*0*^_*ML*_, and *A*^*0*^_*ML*_*(m*_*k*_*, l*_*i*_*)* = *1* if miRNA *m*_*k*_ interacts with lncRNA *l*_*i*_, otherwise, *A*^*0*^_*ML*_*(m*_*k*_*, l*_*i*_*)* = *0.*

#### Construction of known disease-lncRNA association graph

Similarly, let *D* = *{d*_*j*_*; j* = *1,…, n*_*d*_*}* denotes the set of diseases, *L* = *{l*_*i*_*; i* = *1,…,n*_*l*_*}* denotes the set of lncRNAs, where *n*_*d*_*, n*_*l*_ represent number of diseases and lncRNAs, respectively. We can obtain *DL*^*0*^ graph and *A*^*0*^_*DL*_ matrix where *DL*^*0*^ graph is built on known disease-lncRNA associations and *A*^*0*^_*DL*_ is the adjacency matrix of known disease-lncRNA associations. The entity *A*^*0*^_*DL*_*(d*_*j*_*, l*_*i*_*)* is the element in *j*th row and *i*th column of *A*^*0*^_*DL*_, and *A*^*0*^_*DL*_*(d*_*j*_*, l*_*i*_*)* = *1* if disease *d*_*j*_ is associated with lncRNA *l*_*i*_, otherwise, *A*^*0*^_*DL*_*(d*_*j*_*, l*_*i*_*)* = *0*.

#### Construction of a tripartite graph G^0^

From the integration of the three *MD*^*0*^*, ML*^*0*^*, DL*^*0*^ graphs, we obtain a tripartite graph G^*0*^*.* The tripartite graph G^*0*^ is represented by three adjacency matrices: *A*^*0*^_*MD,*_* A*^*0*^_*ML*_ and *A*^*0*^_*DL*_ as mentioned before.

### Construction of a tripartite graph *G*^*u*^

In the tripartite graph G^*0*^, the number of known associations between miRNAs and diseases as well as between miRNAs and lncRNAs are small. So that, for any given lncRNA node *l*_*i*_ and disease node *d*_*j*_, it is clear that the number of miRNA nodes which associated with both *l*_*i*_ and *d*_*j*_ will be very small. To improve it, in our method, we use a collaborative filtering algorithm for recommending suitable miRNA nodes to corresponding lncRNA nodes and disease nodes, respectively. By considering that a recommender system may involve various input data including users and items [18], in our proposed method, we take lncRNAs and diseases as users, while miRNAs as items. For the two adjacency matrices *A*^*0*^_*ML*_ and *A*^*0*^_*MD*_ obtained above, it is easy for us to construct another adjacency matrix *A*^*0*^_*MLD*_ = *[A*^*0*^_*ML*_*, A*^*0*^_*MD*_*]* by splicing *A*^*0*^_*ML*_ and *A*^*0*^_*MD*_ together because the number of rows in both *A*^*0*^_*ML*_ and *A*^*0*^_*MD*_ are same. It is clear that the row vector of *A*^*0*^_*MLD*_ consists of the row vectors in *A*^*0*^_*ML*_ and *A*^*0*^_*MD*_ while the column vectors in *A*^*0*^_*MLD*_ is the same as the column vectors in *A*^*0*^_*ML*_ or *A*^*0*^_*MD*_*.*

On the basis of *A*^*0*^_*MLD*_ and tripartite graph G^*0*^, we can obtain a co-occurrence matrix *R*^*m x m*^, in which, the entity *R(m*_*k*_*, m*_*r*_*)* indicates the element in *k*^*th*^ row and *r*^*th*^ column of *R*^*m x m*^ where *R(m*_*k*_*, m*_*r*_*)* = *1* if and only if the miRNA *m*_*k*_ and miRNA *m*_*r*_ have at least one common neighboring node in *G*^*0*^, otherwise *R(m*_*k*_*, m*_*r*_*)* = *0*. The common neighboring node can be an lncRNA or a disease in *G*^*0*^. So, a similarity matrix *R*^*nor*^ can be calculated by normalizing *R*^*m x m*^ as the following equation:

1 $${\mathrm{R}}^{nor}\left({m}_{k}, {m}_{r}\right)=\frac{\left|N\left({m}_{k}\right)\bigcap N({m}_{r})\right|}{\sqrt{\left|N({m}_{k})\right|*\left|N({m}_{r})\right|}}$$

where *k, r* are the number of miRNAs. $\left|N\left({m}_{k}\right)\right|$ indicates the number of known lncRNAs and diseases associated to *m*_*k*_ in *G*^*0*^, which means the number of elements with value equaling to 1 in *k*th row of *A*^*0*^_*MLD*_. $\left|N\left({m}_{r}\right)\right|$ indicates the number of known lncRNAs and diseases associated to *m*_*r*_ in *G*^*0*^, which means the number of elements with value equaling to 1 in *r*th row of *A*^*0*^_*MLD*_. ∣N(m_k_) ∩ N(m_r_)∣ indicates the number of known lncRNAs and diseases associated with both miRNA *m*_*k*_ and miRNA *m*_*r*_ simultaneously in *G*^*0*^.

Based on the similarity matrix R^nor^ and the adjacency matrix *A*^*0*^_*MLD*_, we calculate a new recommender matrix *A*^*u*^_*MLD*_ as follows:

2 $$A^{u}_{MLD} = \, R^{nor} * \, A^{0}_{MLD}$$

Specifically, for a particular lncRNA *l*_*i*_ or disease *d*_*j*_ in *G*^*0*^, if there is a miRNA *m*_*k*_ satifying *A*^*0*^_*MLD*_*(m*_*k*_*, l*_*i*_*)* = *1* or *A*^*0*^_*MLD*_*(m*_*k*_*, d*_*j*_*)* = *1* in *A*^*0*^_*MLD*_, then we firstly calculate the sum of the values of all elements in the *i*th or *j*th column in *A*^*u*^_*MLD*_, respectively. Therefore, we will have its averaged value ***P***. Next, if the *i*th or *j*th column of *A*^*u*^_*MLD*_ contains a miRNA ${m}_{\theta }$ which satisfies *A*^*u*^_*MLD*_*(*${m}_{\theta }$*, l*_*i*_*)* > ***P**** or A*^*u*^_*MLD*_*(*${m}_{\theta }$*, d*_*j*_*)* > ***P*** then we recommend miRNA ${m}_{\theta }$ for lncRNA *l*_*i*_ or disease *d*_*j*_, respectively. Also, we will add new edge between ${m}_{\theta }$ and *l*_*i*_ or ${m}_{\theta }$ and *d*_*j*_ into the tripartite graph G^*0*^.

Finally, we obtain a tripartite graph *G*^*u*^. The tripartite graph *G*^*u*^ contains three graphs: *MD*^*update*^*, ML*^*update*^ and *DL*^*0*^ and can be represented by three adjacency matrices: *A*^*u*^_*MD*_*, A*^*u*^_*ML*_* and A*^*0*^_*DL*_*. MD*^*update*^ is the updated graph of *MD*^*0*^ after adding new edge between recommended miRNAs and diseases. *ML*^*update*^ is the updated graph of *ML*^*0*^ after adding new edge between recommended miRNAs and lncRNAs. *A*^*u*^_*MD*_ is the adjacency matrix which represents *MD*^*update*^ graph. It contains 10,310 known and recommended associations and 39,850 unknown remained associations. *A*^*u*^_*ML*_ is the adjacency matrix which represents *ML*^*update*^ graph.

### Employing resource allocation process on the tripartite graph *G*^*u*^ to infer miRNA-disease associations

To infer miRNA-disease association, we employ the resource allocation algorithm on the tripartite graph *G*^*u*^ as described in the following steps:

*Step 1*: Calculating resource allocation between miRNAs and diseases

For a specific miRNA m_k_, we define the initial resources located on disease *d*_*j*_ as:

3 $$fd\left( {m_{k} } \right) = A^{u}_{MD} \left( {m_{k} , \, d_{j} } \right),\quad \, j = 1,2, \ldots ,n_{d}$$

where *n*_*d*_ is the number of diseases.

Then we calculate the resource moved back from *D* to *M* by using a weight matrix *W* = *{w*_*kt*_*}n*_*m x*_* n*_*m*_ to indicate the resource allocation process between miRNAs and diseases as follows:

4 $$w_{kt} = \frac{1}{{\deg A_{MD}^{u} \left( {m_{k} } \right)}}*\mathop \sum \limits_{j = 1}^{{n_{d} }} \frac{{A_{MD }^{u} \left( {m_{k} , d_{j} } \right) * A_{MD }^{u} \left( {m_{t} , d_{j} } \right)}}{{\deg A_{MD}^{u} \left( {d_{j} } \right)}}$$

where ${w}_{kt}$ is the contribution resource moved from *t*th node to *k*th node in *M*, and it can be understood as the similarity between miRNA *m*_*k*_ and miRNA *m*_*t*_ in *MD*^*update*^ graph. $\mathit{deg}{A}_{MD}^{u}\left({m}_{k}\right)$ is the degree of miRNA *m*_*k*_ in *MD*^*update*^ graph and it represents the number of associated diseases for miRNA *m*_*k*_. Similarly, $\mathit{deg}{A}_{MD}^{u}\left({d}_{j}\right)$ is the degree of disease *d*_*j*_ in *MD*^*update*^ graph and it represents the number of associated miRNAs for disease *d*_*j*_.

With respect to previous study [20], we also modify the resource allocation algorithm by considering the level of consistency between the contribution of resource transferred in both directions. It shows the impact of co-selection *(m*_*k*_*, m*_*t*_*)* between the contribution of resource from *m*_*k*_ to *m*_*t*_ and the contribution of resource from *m*_*t*_ to *m*_*k*_. A consistence-based resource allocation to represent a final miRNA-disease weight matrix *W’* = *{w’*_*kt*_*}* can be defined as in the following equation:

5 $$W_{kt}^{^{\prime}} = W_{kt} + \frac{{W_{tk} }}{{\mathop \sum \nolimits_{s = 1}^{{n_{m} }} W_{sk} }}$$

From the combination of the final miRNA-disease weight matrix *W’* and the adjacency matrix *A*^*u*^_*MD*_, we define a final resource *Rscore_ondisease_1* located on *D* as follows:

6 $$Rscore\_ondisease\_1 = W^{{\prime }} *A^{u}_{MD}$$

*Step 2*: Calculating resource allocation between diseases and lncRNAs

In regard to resource allocation between genes and diseases in TPGLDA [20], the same initial resources located on *M* nodes are allocated from nodes in *M* to nodes in *D* and then moved back, and the final resource matrix *Rscore_ondisease_2* located on *D* nodes are issued by:

7 $$Rscore\_ondisease\_2 = \mathop \sum \limits_{s = 1}^{{n_{l} }} \frac{{A_{DL }^{0} \left( {d_{j} , l_{s} } \right) }}{{\deg A_{DL}^{0} \left( {l_{i} } \right)}}*\mathop \sum \limits_{k = 1}^{{n_{d} }} \frac{{A_{MD}^{u} \left( { m_{k} , d_{j} } \right)}}{{\deg A_{DL}^{0} \left( {d_{j} } \right)}}$$

where $\mathrm{deg}{A}_{DL}^{0}\left({l}_{i}\right)={\sum }_{j=1}^{{n}_{d}}{A}_{DL}^{0}({d}_{j}, {l}_{i})$ is the number of related diseases for lncRNA *l*_*i*_ or the degree of lncRNA *l*_*i*_ in *DL*^*0*^ graph. $\mathrm{deg}{A}_{DL}^{0}\left({d}_{j}\right)$=${\sum }_{i=1}^{{n}_{l}}{A}_{DL}^{0}({d}_{j}, {l}_{i})$ is the number of related lncRNAs for disease *d*_*j*_ or the degree of disease *d*_*j*_ in *DL*^*0*^ graph.

*Step 3*: Calculating the final resource score *Rscore_final* to infer the potential disease-related miRNAs

We calculate the final resource score *Rscore_final* which is used to measure latent disease-related miRNAs as follows:

8 $$Rscore\_final = \gamma * Rscore\_ondisease\_1 + \, \left( {1 - \gamma } \right) \, *Rscore\_ondisease\_2$$

where *γ* is a tunable parameter with value in [0, 1]. Our model achieves the best prediction performance when *γ* = 0*.9.*

### Ranking all candidate miRNAs’ Rscores for each disease in descending order

Finally, we sort all candidate miRNAs’ *Rscore_final* for each disease in descending order so that a higher score candidate will have more chances to be verified in the future.

## Results

### Performance measures

To evaluate our method performance in inferring miRNA-disease associations, we performed the fivefold-cross-validation experiments and evaluated the Area under roc curve (AUC) and the Area under precision-recall curve (AUPR) as described in following sections:

### Evaluating the AUC under 5-fold-cross validation

**Fig. 2:**
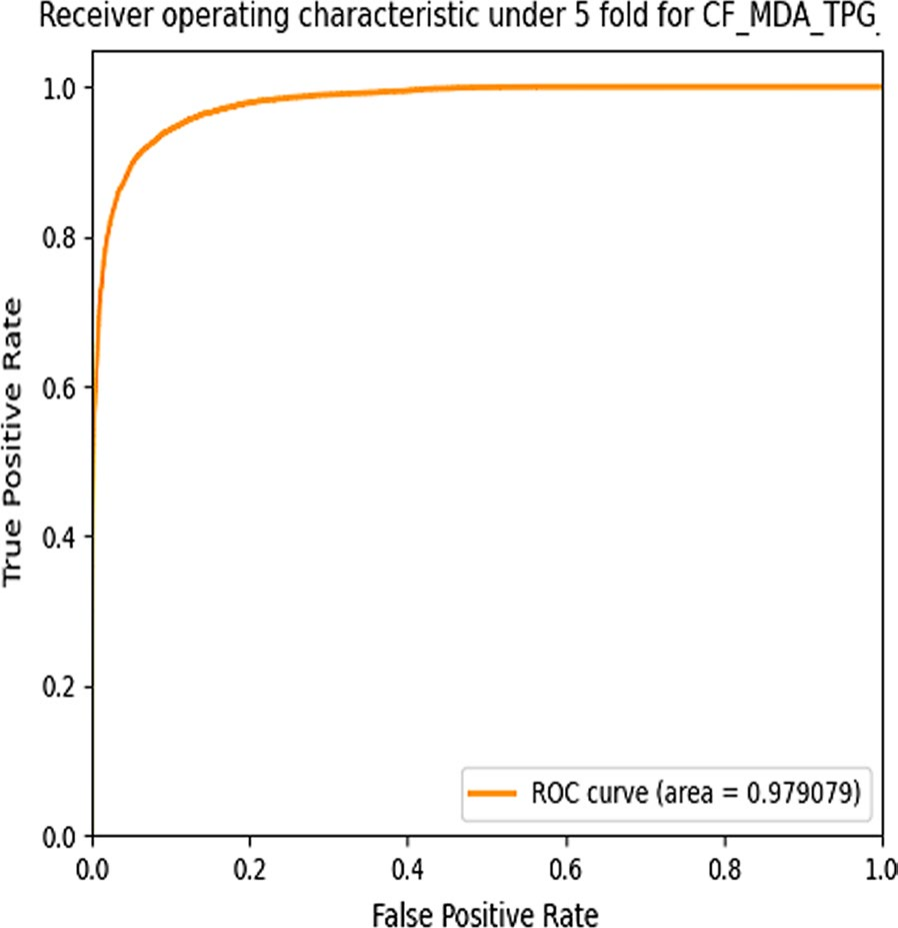
AUC curve with *γ* = 0.9 in one experimental running time

After applying a collaborative filtering algorithm on tripatite graph G^*0*^, we obtained a tripartite graph *G*^*u*^ which contained three subgraphs: *MD*^*updated*^ graph, *ML*^*updated*^ graph and *DL*^*0*^ graph. By employing the resource allocation algorithm on the tripartite graph *G*^*u*^, we predicted potential miRNA-disease associations. To evaluate our model performance in AUC term [26], we compared the inferred miRNA-disease associations resulted in *Rscore_final* matrix with the adjacency matrix *A*^*u*^_*MD*_ of *MD*^*updated*^ graph. In *MD*^*updated*^ graph, we considered 10,310 associations of known and recommended associations as positive samples and the 39,850 remained unknown associations as negative samples. Then we randomly divided all positive and negative samples into 5 equal parts to perform fivefold-cross-validation. Next, in each running time, we used 4 parts of positive and negative samples for training and the remain part for testing. Our model is trained to recalculate *Rscore_final* in each running time. Basically, we computed the false positive rate (FPR) and true positive rate (TPR) with different *γ* values where FPR indicates the proportion of the real negative samples in predicted positive samples to all negative samples and TPR indicates the proportion of the real positive samples in all predicted positive samples. The FPR and TPR are calculated by the following equations:

9 $$FPR=\frac{FP}{FP+TN}$$

10 $$TPR= \frac{TP}{TP+FN}$$

where TP (true positive) means that a positive sample is correctly predicted as positive sample; FN (false negative) means that a positive sample is incorrectly predicted as negative sample; FP (false positive) indicates that a negative sample incorrectly predicted as positive sample; TN (true negative) indicates that a negative sample is correctly predicted as negative sample. We use TPR as vertical axis and FPR as horizontal axis to draw the receiver operating characteristic (ROC) curve [32], and the AUC value of our model achieves 0.9788 with *γ* = 0*.*9 after we perform the experiment for 10 times under fivefold-cross-validation. Figure 2 illustrates AUC curve with *γ* = 0.9 in one experimental running time.

### Evaluate AUPR under 5-fold-cross validation

As previously mentioned, the data to evaluate our model performance is not balanced. Therefore, we also draw precision-recall curve and calculate the AUPR curve to evaluate prediction performance [27]. The Precision reflects the percentage of the accurately predicted positive samples in all predicted positive samples, and the Recall reflects the percentage of the accurately predicted positive samples in all real positive samples. We calculate Precision and Recall as follows:

11 $$Precision=\frac{TP}{TP+FP}$$

12 $$Recall=\frac{TP}{TP+FN}$$

**Fig. 3:**
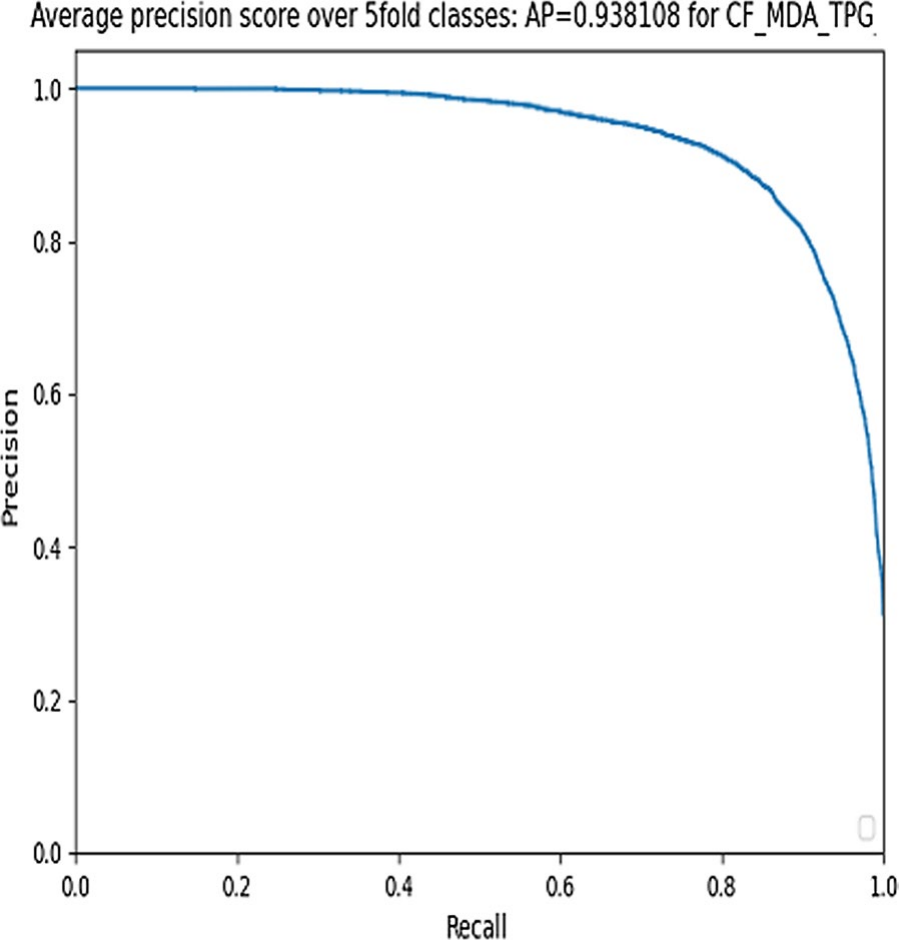
AUPR curve with *γ* = 0.9 in one experimental running time

After we perform the experiment under fivefold-cross-validation for 10 times, our model achieves the best AUPR value 0.9373 with *γ* = 0.9. Figure 3 illustrates AUPR curve with *γ* = 0.9 one experimental running time.

### Performance comparison with other related models

To demonstrate the outperformance of our model, we compare our model performance with the performance of DCSMDA method proposed by Zhao et al*.* [7]**.** We also implements predicting miRNA-disease associations by applying the resource allocation process introduced in [20] without applying collaborative filtering algorithm. The performances of these methods are shown in the Table 1.

**Table 1:** Performance comparison with other related models

Method	AUC value	AUPR value
TPGLDA	0.9703	0.7421
DCSMDA	0.8155	–
Our model	0.9788	0.9373

As can be seen, our proposed method achieves better performance in comparison with DCSMDA and the method of applying TPGLDA in prediction of miRNA-disease associations for both AUC and AUPR values. Because of the sparsity data problem, AUC value usually achieves high score. However, in our proposed method, by using collaborative filtering algorithm to improve the density of miRNA-disease associations so that the updated adjacency matrix *A*^*u*^_*MD*_ becomes more balanced which implies that the AUPR value (0.9373) could significantly be improved in comparison to AUPR value (0.7421) in case of applying TPGLDA model to predict miRNA-disease associations without using collaborative filtering algorithm. It demonstrates that our model achieves a more reliable performance than other previous methods.

### Case studies

In addition to fivefold-cross-validation experiments, we also employed some case studies on our proposed model by sorting all candidate miRNAs for each disease. These predictions are utilized for further validation. In consistence with the previous study [20], all known and recommended miRNA-disease associations are considered as training samples, then the *Rscore_final* for each potential miRNA-disease association is calculated in sequence. Higher *Rscore_final* value indicates greater potential miRNA-disease association. In more detail, case studies on Prostatic Neoplasms, Heart Failure, Glioma and Open-angle Glaucoma are constructed to show the ability of our model in order to identify new disease-associated miRNAs.

*Prostatic neoplasms*, also known as Prostate Cancer, is the second-most prevalent type of cancers and the fifth-leading cause of cancer-related death in men [28]. miRNAs have been shown to play an important role in predicting prognosis of Prostate Cancer. Up to now, a variety of miRNAs have been reported to be associated with Prostatic Neoplasms /Prostate Cancer. For example, a target gene of miR-653-5p represses the proliferation and invasion of prostate cancer cells [29]. The dual action of miR-125b as a Tumor Suppressor and OncomiR-22 promotes Prostate Cancer tumorigenesis [30]. As shown in Table 2, there are 8 new miRNA-disease associations out of top predicted 40 miRNAs by applying our proposed method. All of new 8 miRNA-disease associations were confirmed by recent literatures.

**Table 2:** Top 40 predicted miRNAs for Prostatic Neoplasms

miRNA	Rank	Known before	Evidence (PMID)	miRNA	Rank	Known before	Evidence (PMID)
hsa-mir-20a	1	1	20944140	hsa-mir-10b	21	0	28320379
hsa-mir-34a	2	1	25032850	hsa-mir-31	22	1	23233736
hsa-mir-146a	3	1	27222754	hsa-mir-34c	23	1	28320379
hsa-mir-17	4	1	30001402	hsa-mir-224	24	1	30542718
hsa-mir-125b	5	1	28320379	hsa-mir-9	25	0	28320379
hsa-mir-21	6	1	27699004	hsa-mir-19a	26	0	29138858
hsa-mir-92a	7	1	28320379	hsa-mir-486	27	1	27877055
hsa-mir-155	8	0	28320379	hsa-mir-146b	28	1	32368293
hsa-mir-145	9	1	28320379	hsa-mir-183	29	1	23538390
hsa-mir-182	10	1	28320379	hsa-mir-124	30	1	28320379
hsa-mir-200c	11	1	31157262	hsa-mir-148a	31	1	28320379
hsa-mir-27a	12	1	31258791	hsa-mir-181a	32	0	32197476
hsa-mir-218	13	1	28030804	hsa-mir-30c	33	1	28320379
hsa-mir-200b	14	1	28320379	hsa-mir-221	34	1	24892674
hsa-mir-18a	15	0	28320379	hsa-mir-16	35	1	28320379
hsa-mir-126	16	1	29805636	hsa-let-7d	36	1	31468250
hsa-mir-101	17	1	21430074	hsa-let-7b	37	1	27157642
hsa-mir-143	18	1	30933831	hsa-let-7c	38	1	28320379
hsa-mir-25	19	1	28320379	hsa-mir-210	39	0	27824162
hsa-let-7a	20	1	28320379	hsa-mir-196a	40	0	28982366

*Heart failure (HF)*, also known as congestive heart failure (CHF) and congestive cardiac failure (CCF), is when the heart is unable to pump sufficiently to maintain blood flow to meet the body's needs. It is a widely prevalent syndrome imposing a significant burden of morbidity and mortality world-wide [31]. Unravelling the functional relevance of miRNAs within pathogenic pathways is a major challenge in cardiovascular research. Recently, a numerous miRNAs have been reported to be associated with heart failure. For instance, plasma miR-126 levels are up-regulated in HF patients [32]. MicroRNA-34 family members (miR-34a, -34b, and -34c) are up-regulated in the heart in response to stress [33]. Local microRNA-133a downregulation is associated with hypertrophy in the dyssynchronous heart [34]. Table 3 shows top 40 predicted heart failure related miRNAs by applying our proposed method. As can be seen, it contains 19 new miRNAs associated with Heart failure**.** All of these predicted associations were confirmed by literatures.

**Table 3:** Top 40 predicted miRNAs for Heart failure

miRNA	Rank	Known before	Evidence (PMID)	miRNA	Rank	Known before	Evidence (PMID)
hsa-mir-20a	1	0	27173194	hsa-mir-101	21	0	17712037
hsa-mir-125b	2	0	23736534	hsa-let-7a	22	0	23736534
hsa-mir-21	3	1	30783473	hsa-mir-18a	23	1	28293796
hsa-mir-34a	4	0	28660188	hsa-mir-224	24	0	23736534
hsa-mir-146a	5	0	30355233	hsa-mir-9	25	0	23736534
hsa-mir-155	6	1	30783473	hsa-mir-19a	26	1	20118173
hsa-mir-17	7	1	27058529	hsa-mir-486	27	0	26485305
hsa-mir-182	8	1	25013816	hsa-mir-124	28	0	23736534
hsa-mir-92a	9	1	23736534	hsa-mir-146b	29	0	20118173
hsa-mir-126	10	1	29062343	hsa-mir-148a	30	1	23736534
hsa-mir-145	11	1	30783473	hsa-mir-183	31	0	27544699
hsa-mir-34c	12	1	30988323	hsa-mir-181a	32	1	30783473
hsa-mir-200c	13	1	23736534	hsa-mir-30c	33	1	30783473
hsa-mir-27a	14	1	22136461	hsa-let-7d	34	0	20118173
hsa-mir-218	15	1	20118173	hsa-mir-16	35	1	20118173
hsa-mir-25	16	0	30783473	hsa-mir-221	36	0	30009269
hsa-mir-200b	17	0	23864135	hsa-let-7b	37	0	20118173
hsa-mir-10b	18	1	30783473	hsa-let-7c	38	0	23736534
hsa-mir-31	19	0	20118173	hsa-mir-210	39	1	31249,644
hsa-mir-143	20	0	30783473	hsa-mir-191	40	0	20118173

*Glioma* is the most common central nervous system tumor and associated with poor prognosis. Identifying effective diagnostic biomarkers for glioma is particularly important in order to guide optimizing treatment [35]. Many studies have shown that some miRNAs are correlated with the diagnosis and prognosis of gliomas. For example, MiR-34a acts as tumor-suppressor by targeting many oncogenes related to proliferation, apoptosis, and invasion of gliomas [36]. MicroRNA (miR) 125b regulates cell growth and invasion in pediatric low grade glioma [37]. MicroRNA-21 promotes migration and invasion of glioma cells via activation of Sox2 and β-catenin signaling [38]. Therefore, in this study, we chose glioma as a case study to demonstrate our model’s ability in prediction associations between miRNAs and diseases. Table 4 lists top 40 glioma associated miRNAs inferred by our model. As illustrated, there are 14 new miRNAs associated with glioma, which are uncovered by applying our proposed method and all of them have been validated by literatures.

**Table 4:** Top 40 predicted miRNAs for Glioma

miRNA	Rank	Known before	Evidence (PMID)	miRNA	Rank	Known before	Evidence (PMID)
hsa-mir-20a	1	1	27123147	hsa-mir-34c	21	0	24179539
hsa-mir-17	2	1	30524906	hsa-mir-25	22	0	27123147
hsa-mir-125b	3	1	30131528	hsa-let-7a	23	0	24092860
hsa-mir-21	4	1	22468222	hsa-mir-224	24	1	31046428
hsa-mir-34a	5	1	30836600	hsa-mir-19a	25	0	29340016
hsa-mir-146a	6	0	22468222	hsa-mir-9	26	1	22468222
hsa-mir-92a	7	0	27801803	hsa-mir-486	27	0	32094299
hsa-mir-155	8	0	24376632	hsa-mir-181a	28	1	18710654
hsa-mir-182	9	1	20472885	hsa-mir-146b	29	1	30018734
hsa-mir-145	10	1	23814265	hsa-mir-124	30	1	22468222
hsa-mir-18a	11	1	28123848	hsa-mir-148a	31	0	28445981
hsa-mir-200c	12	0	30034253	hsa-mir-183	32	1	23263745
hsa-mir-27a	13	1	25628931	hsa-mir-16	33	1	28628119
hsa-mir-218	14	1	28431179	hsa-mir-221	34	1	31180529
hsa-mir-126	15	0	29633591	hsa-mir-30c	35	0	29495977
hsa-mir-200b	16	1	30034253	hsa-let-7d	36	0	31868319
hsa-mir-143	17	1	24980823	hsa-mir-93	37	1	27185265
hsa-mir-10b	18	1	28431179	hsa-mir-196a	38	1	24463357
hsa-mir-31	19	1	29521593	hsa-mir-214	39	1	29234674
hsa-mir-101	20	0	21321380	hsa-mir-181b	40	1	18710654

*Glaucoma* is the second leading cause of blindness in the United States of America [39]. The most common types of open-angle glaucoma (OAG) are primary open-angle glaucoma (POAG) and exfoliation glaucoma (XFG) [40]. Recent studies have shown that miRNAs may play a role in pathways implicated in glaucoma and act as biomarkers for disease pathogenesis [41]. In this paper, open-angle glaucoma is considered as an isolated disease because it is not associated with any miRNAs in the used datasets. However, our proposed method can be used to discover new associations for new diseases (or miRNAs) without any known associations before. As illustrated in Table 5, by applying our proposed method, 11 out of top 20 predicted open-angle glaucoma-related miRNAs have been confirmed by recent literatures.

**Table 5:** Top 20 miRNAs for GlaucomaOpen-Angle

miRNA	Rank	Known before	Evidence (PMID/reference)
hsa-mir-20a	1	0	Unknown
hsa-mir-125b	2	0	29401312
hsa-mir-21	3	0	29401312
hsa-mir-34a	4	0	Reference [42]
hsa-mir-146a	5	0	Unknown
hsa-mir-155	6	0	29401312
hsa-mir-126	7	0	31153869
hsa-mir-25	8	0	Unknown
hsa-mir-34c	9	0	Unknown
hsa-mir-145	10	0	28424493
hsa-mir-182	11	0	27537254
hsa-mir-17	12	0	32178636
hsa-mir-200c	13	0	30025119
hsa-mir-27a	14	0	32178636
hsa-mir-218	15	0	Unknown
hsa-mir-92a	16	0	Unknown
hsa-mir-143	17	0	30025119
hsa-mir-10b	18	0	Unknown
hsa-mir-31	19	0	Unknown
hsa-mir-101	20	0	Unknown

## Discussions

Although our proposed method achieved a reliable performance, it still exists some limitations which require further research. Firstly, our method still focuses on unweighted tripartite graph, so it may be improved by weighting the known lncRNA-disease associations, known miRNA-disease associations, and verified lncRNA-miRNA interactions. Secondly, enhancing the algorithm of appropriating resources can integrate the updated lncRNA-miRNA interactions into resource allocation process. Finally, the latest useful datasets should be collected to update our dataset library (Additional files 1, 2, 3, 4, 5).

## Conclusion

In this paper, we proposed a new method to infer miRNA-disease associations using collaborative filtering and resource allocation on a miRNA-disease-lncRNA tripartite graph. By applying our proposed method, we can improve prediction accuracy, solve the sparsity data problem, and have not to use subjective and not directly related to association prediction information. The experimental results show that our method achieves a reliable performance with AUC and AUPR values 0.9788 and 0.9373, respectively, which is more impressive than several mentioned previously methods. It demonstrates the ability to infer new associations between miRNAs and diseases as indicated in case studies of Prostatic Neoplasms, Heart Failure, and Glioma diseases. Besides, it can discover new associations for new diseases (or miRNAs) without any known associations as indicated in the case study of Open-angle glaucoma disease. It suggests that our method can be considered as a powerful tool to predict miRNA-disease associations.

## Supplementary Information

**Additional file 1**: For known lncRNA-disease associations.

**Additional file 2**: For known lncRNA-disease associations.

**Additional file 3**: For known lncRNA-disease associations.

**Additional file 4**: For known lncRNA-miRNA associations.

**Additional file 5**: For known disease-miRNA associations.

## Data Availability

The datasets used in our research were collected from Zhao et al.’s study https://doi.org/10.1186/s12859-018-2146-x [7].

## Abbreviations

AUC: Area under Roc curve
AUPR: Area under precision-recall curve
FN: False negative
FP: False positive
FPR: False positive rate
TP: True positive
TPR: True positive rate
lncRNA: Long non-coding RNA
miRNA: Micro RNA
OAG: Open-angle glaucoma
POAG: Primary open-angle glaucoma
